# Comparative in vitro toxicity of a graphene oxide-silver nanocomposite and the pristine counterparts toward macrophages

**DOI:** 10.1186/s12951-016-0165-1

**Published:** 2016-02-24

**Authors:** Luis Augusto Visani de Luna, Ana Carolina Mazarin de Moraes, Sílvio Roberto Consonni, Catarinie Diniz Pereira, Solange Cadore, Selma Giorgio, Oswaldo Luiz Alves

**Affiliations:** Laboratory of Solid State Chemistry (LQES), Institute of Chemistry, University of Campinas, Campinas, Brazil; Brazilian Biosciences National Laboratory (LNBio), Brazilian Center for Research in Energy and Materials (CNPEM), Campinas, Brazil; Laboratory of Cytochemistry and Immunocytochemistry (LCI), Institute of Biology, University of Campinas, Campinas, Brazil; Laboratory of Leishmaniasis (Lableish), Institute of Biology, University of Campinas, Campinas, Brazil; Atomic Spectrometry Group (GEAtom), Institute of Chemistry, University of Campinas, Campinas, Brazil

**Keywords:** Graphene oxide-silver nanocomposite, Macrophage, Internalization, Reactive oxygen species, Synergism

## Abstract

**Background:**

Graphene oxide (GO) is a highly oxidized graphene form with oxygen functional groups on its surface. GO is an excellent platform to support and stabilize silver nanoparticles (AgNP), which gives rise to the graphene oxide-silver nanoparticle (GOAg) nanocomposite. Understanding how this nanocomposite interacts with cells is a toxicological challenge of great importance for future biomedical applications, and macrophage cells can provide information concerning the biocompatibility of these nanomaterials. The cytotoxicity of the GOAg nanocomposite, pristine GO, and pristine AgNP was compared toward two representative murine macrophages: a tumoral lineage (J774) and peritoneal macrophages collected from Balb/c mouse. The production of reactive oxygen species (ROS) by J774 macrophages was also monitored. We investigated the internalization of nanomaterials by transmission electron microscopy (TEM). The quantification of internalized silver was carried out by inductively coupled plasma mass spectrometry (ICP-MS). Nanomaterial stability in the cell media was investigated overtime by visual observation, inductively coupled plasma optical emission spectrometry (ICP OES), and dynamic light scattering (DLS).

**Results:**

The GOAg nanocomposite was more toxic than pristine GO and pristine AgNP for both macrophages, and it significantly induced more ROS production compared to pristine AgNP. TEM analysis showed that GOAg was internalized by tumoral J774 macrophages. However, macrophages internalized approximately 60 % less GOAg than did pristine AgNP. The images also showed the degradation of nanocomposite inside cells.

**Conclusions:**

Although the GOAg nanocomposite was less internalized by the macrophage cells, it was more toxic than the pristine counterparts and induced remarkable oxidative stress. Our findings strongly reveal a synergistic toxicity effect of the GOAg nanocomposite. The toxicity and fate of nanocomposites in cells are some of the major concerns in the development of novel biocompatible materials and must be carefully evaluated.

**Electronic supplementary material:**

The online version of this article (doi:10.1186/s12951-016-0165-1) contains supplementary material, which is available to authorized users.

## Background

Recent advances in nanotechnology have greatly increased the biological applications of nanomaterials, predominantly in the field of nanomedicine. Since the discovery of carbon nanotubes, no other carbon nanomaterial has attracted as much attention in the scientific community as graphene. Graphene consists of bidimensional sheets of carbon atoms arranged in hexagonal rings [1–3]. Its highly oxidized form is the so-called graphene oxide (GO), which is characterized by the presence of oxygen-containing moieties, such as epoxy, hydroxyl, carbonyl and carboxyl groups, on the basal plane and edges of the sheets [4, 5]. Graphene-based nanomaterials have been used in a range of biological applications, including biosensors because of their preferential interactions with single strand DNA, bioimaging tools because of their intrinsic fluorescence and/or facile functionalization with fluorophores, carrier of genes for cellular transfection, delivery of small molecules of drugs for cancer treatment, and scaffolds for mammalian cell proliferation and differentiation [6–8].

Graphene also represents a valuable platform for the development of nanocomposites, allowing the combination of nanomaterials with different properties to give novel materials with improved or new functionalities. Specifically, GO is an important platform for the attachment of silver nanoparticles. The high surface area of GO sheets serves as a support for growth and stabilization of nanoparticles [9], which prevents them from aggregating. Moreover, as these silver-based nanocomposites have excellent antimicrobial properties [10–14], they represent an alternative to the inefficacies of long-used antibiotics.

Graphene toxicity has been studied by several groups owing to its distinct physicochemical characteristics such as purity, lateral dimension [15], size of the sheets [16], and oxidation-state [17], which may influence its cellular uptake, biodegradation and toxicity. Once in contact with the cell membrane, graphene sheets can create an impermeable encasement affecting the normal exchange between the cell and the extracellular environment [18], Graphene oxide can also damage the cell membrane through strong electrostatic interactions between the negatively charged oxygen groups on its surface and the positively charged lipids present on cell membranes [19].

In general, graphene oxide can be internalized by cells through endocytic mechanisms, such as macropinocytosis and clathrin-dependent pathways [20]. However, graphene can spontaneously penetrate the plasma membrane and it can be found freely localized in the cytosol interacting with cellular organelles, such as lysosomes and mitochondria [21, 22]. Inside the cells, graphene leads to increased oxidative stress and metabolic activity associated with repair mechanisms [23]. Particularly for macrophage cells, graphene can activate the membrane Toll-Like receptor 4 (TLR4) and induce necrotic death. However, when inside the macrophage, cytoskeletal damage and oxidative stress may also be related to the decrease in macrophage viability [23].

Silver nanoparticles (AgNP) have been extensively explored as a biocidal agent and their toxicity mechanisms are associated with cell membrane damage and oxidative stress [24, 25]. Silver ions bind to protein disulfide bonds in the cytoplasm, causing deformities in the protein structure. These malformed proteins are then incorporated into the plasma membrane, leading to alterations in cell permeability and cellular death [26]. Moreover, silver dysregulates the mitochondrial respiratory chain and reduces the efficiency of antioxidant enzymes such as glutathione transferase, resulting in the overproduction of free radicals [27]. The toxicity of AgNP may also be related to other mechanisms, such as the inhibition of DNA synthesis [28], actin depolymerization, membrane instability, and intracellular calcium overload, all of which induce early cell apoptosis [29]. Although the toxicity mechanisms of pristine nanomaterials, such as graphene oxide and silver nanoparticles, have been widely exploited, studies related to the toxicity of graphene-silver nanocomposites are still scarce in the literature.

Several cellular models have been used to assess the toxicity of nanomaterials; however, macrophage cells are common targets in studies addressing the biocompatibility of novel materials. Macrophages are part of the cell-mediated immune system and originate from bone-marrow monocytes that migrate through the circulation and cross the endothelium to reach the tissues. The monocytes differentiate into macrophages that adopt specialized phenotypes to tackle infections and recruit other immune cells. Macrophages are versatile and can be found playing multiple functions in every tissue, thus acting as antigen presenters and producing a range of biologically active substances such as cytokines, tumoral growth factors, angiogenesis factors, coagulation factors, interferons, and enzymes with high hydrolytic activity and free radicals, such as nitric oxide and superoxide [30, 31]. Macrophages also exhibit phagocytic activity, an energy-dependent internalization mechanism. In this process, the cell membrane can enclose microorganisms and particles, forming vesicles and vacuoles for biodegradation [32, 33]. In this way, nanomaterials can end up in those cells [22], which may result in toxicity.

In this sense, the interaction of nanomaterials with macrophages has been closely studied to understand the toxicity outcomes. For instance, Wang et al. observed a cell-type, dose-, and time-dependent production of reactive oxygen species (ROS) in A549 lung epithelial cells and J774 macrophages after exposure to commercial nanomaterials such as carbon nanotubes, aluminum oxide, titanium dioxide, and silver nanoparticles. The authors reported a correlation between ROS production and cellular death after observing that increased oxidative stress leads to a reduction in macrophage viability [34].

In addition to toxicity, nanomaterials may also elicit changes in macrophage behavior. Macrophage modulation after exposure to a nanocomposite was observed by Taylor et al., who demonstrated through in vitro experiments that human macrophages (THP-1) internalized carbon nanotubes coated with aluminum oxide, which altered the production of cytokines related to the inflammatory response [35]. The internalization and biodegradation of carbon nanohorns was investigated by Zhang et al. through in vitro experiments with RAW 264.7 and THP-1 macrophages. The authors observed that after 9 days, 30 % of the internalized nanomaterial was degraded by those cells. They also found that macrophages produce ROS in response to nanomaterial exposure, thus highlighting the importance of these radicals in the degradation of carbon nanohorns [32].

In this study, we performed in vitro experiments to assess the toxicity of a graphene oxide-silver nanocomposite and its counterparts, pristine graphene oxide and pristine silver nanoparticles toward murine tumoral macrophages from cell lineage J774 and primary macrophages collected from mouse peritoneum. We also investigated the induction of oxidative stress by these nanomaterials and their internalization by J774 tumoral macrophages. The GOAg nanocomposite was found to be more toxic toward macrophage cells than its pristine counterparts. Additionally, the nanocomposite was less internalized by the tumoral cell than pristine AgNP. To the best of our knowledge, this is the first report addressing the cytotoxicity of graphene oxide-silver nanocomposite toward macrophages. Given the emerging field of nanomedicine, assessing the biocompatibility of nanomaterials using macrophage cells is a toxicological issue of major importance for future biomedical applications.

## Results and discussion

### Characterization of nanomaterials

**Fig. 1:**
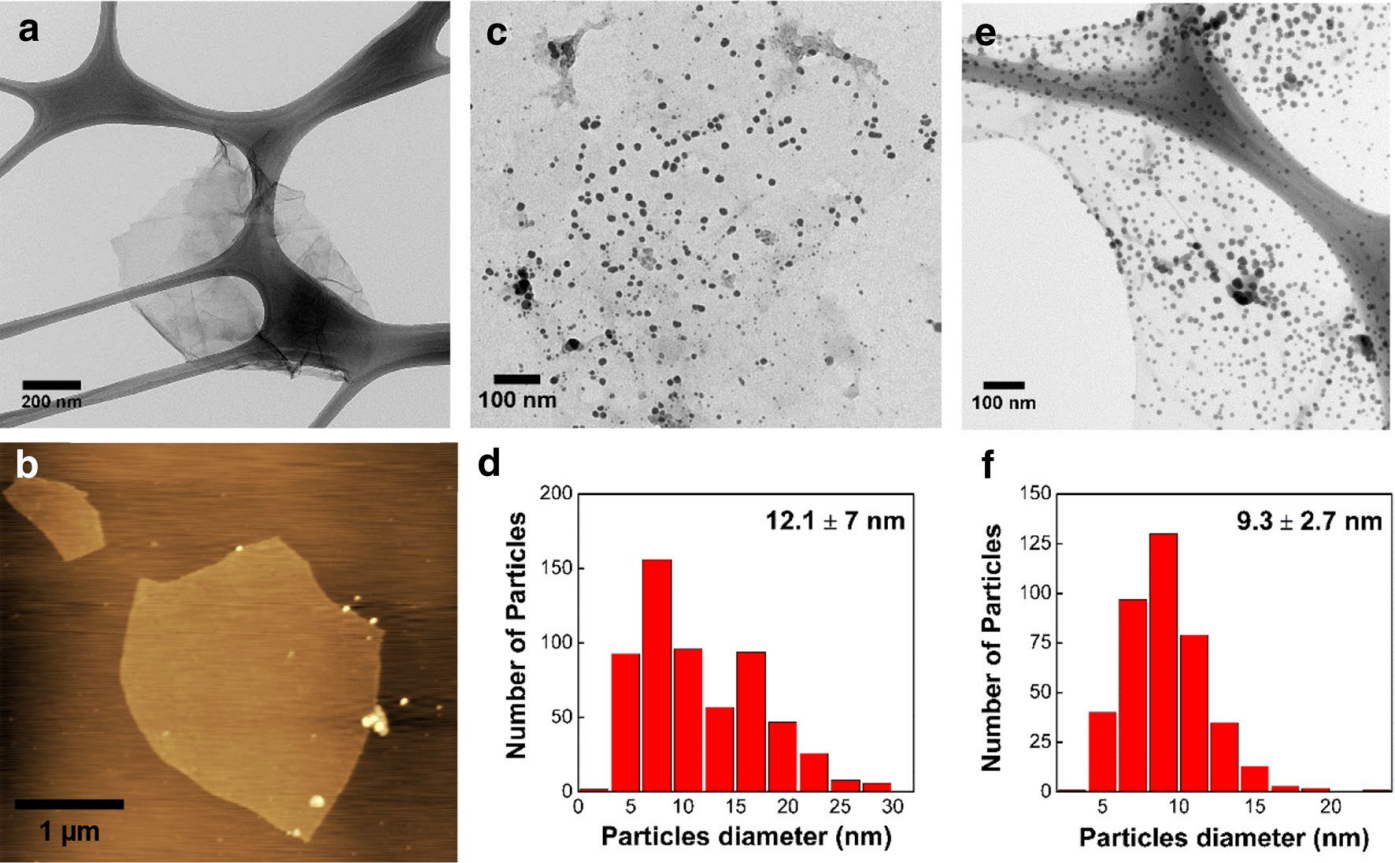
TEM images of nanomaterials and their size distribution. **a** TEM and **b** AFM images of pristine graphene oxide. **c** TEM image and **d** size distribution of pristine silver nanoparticles. **e** TEM image of graphene oxide-silver nanocomposite. **f** Size distribution of silver nanoparticles attached to GO sheets

The formation of pristine AgNP and the attachment of AgNP on the GO surface were primarily confirmed through the detection of the plasmon resonance band at 415 and 410 nm, respectively (Additional file 1: Figure S1). The X-ray diffraction analysis of the pristine AgNP and GOAg nanocomposite shows the crystalline planes of face-centered cubic silver nanoparticles (Additional file 2: Figure S2). The morphology of GO, AgNP, and GOAg was investigated by transmission electron microscopy (TEM). Figure 1a shows a transparent and stable GO sheet. The sheets also depicts lateral dimensions in micrometers and a thickness of approximately 1 nm as observed by atomic force microscopy imaging (AFM) (Fig. 1 b). The pristine citrate-stabilized AgNP exhibited spherical-like morphology, with an average size of 12.1 ± 7.0 nm (Fig. 1c, d). Similarly, the AgNP attached to the GO surface were spherical and well dispersed throughout the sheets and presented no evidence of agglomeration (Fig. 1e). In addition, the AgNP were exclusively supported on GO sheets and non-attached nanoparticles were not observed. The nanocomposite exhibited AgNP with an average size of 9.3 ± 2.7 nm (Fig. 1f).

Prior the cytotoxicity tests, the concentration of silver was determined by inductively coupled plasma optical emission spectrometry (ICP OES). Stock dispersions of AgNP and GOAg contained 146.1 and 121.0 µg mL^−1^ of silver, respectively (Table 1). This determination implies that the GO:Ag mass ratio in the nanocomposite was approximately 1, because the silver concentration is practically the same as the initial concentration of the GO dispersion (120.0 µg mL^−1^).

**Table 1:** Silver concentration and zeta potential of nanomaterials

Stock solution	ICP OES		Zeta potential		
	[Ag] µg mL^−1^	[GO] µg mL^−1^	ζ (mV)		
			DI	RPMI	RPMI + FBS
GO	–	120.0	−44.8	−11.5	−12.3
AgNP	146.1	–	−38.3	−14.9	−13.3
GOAg	121.0	120.0	−49.4	−22.4	−0.32

Zeta potential measurements demonstrated that all nanomaterials were negatively charged regardless the dispersion media (Table 1). The GOAg nanocomposite exhibited the greatest negative potential (−49.4 mV) when dispersed in deionized water (DI) (Table 1). The negatively charged surface of pristine AgNP may be explained by the stabilization of nanoparticles by citrate anions, whereas the GO sheets are negatively charged owing to the presence of oxygenated surface moieties, such as hydroxyl, epoxy, and carbonyl groups. Additional characterization of GO is available in a previous work by our group [36].

### Nanomaterials stability in cell media

**Fig. 2:**
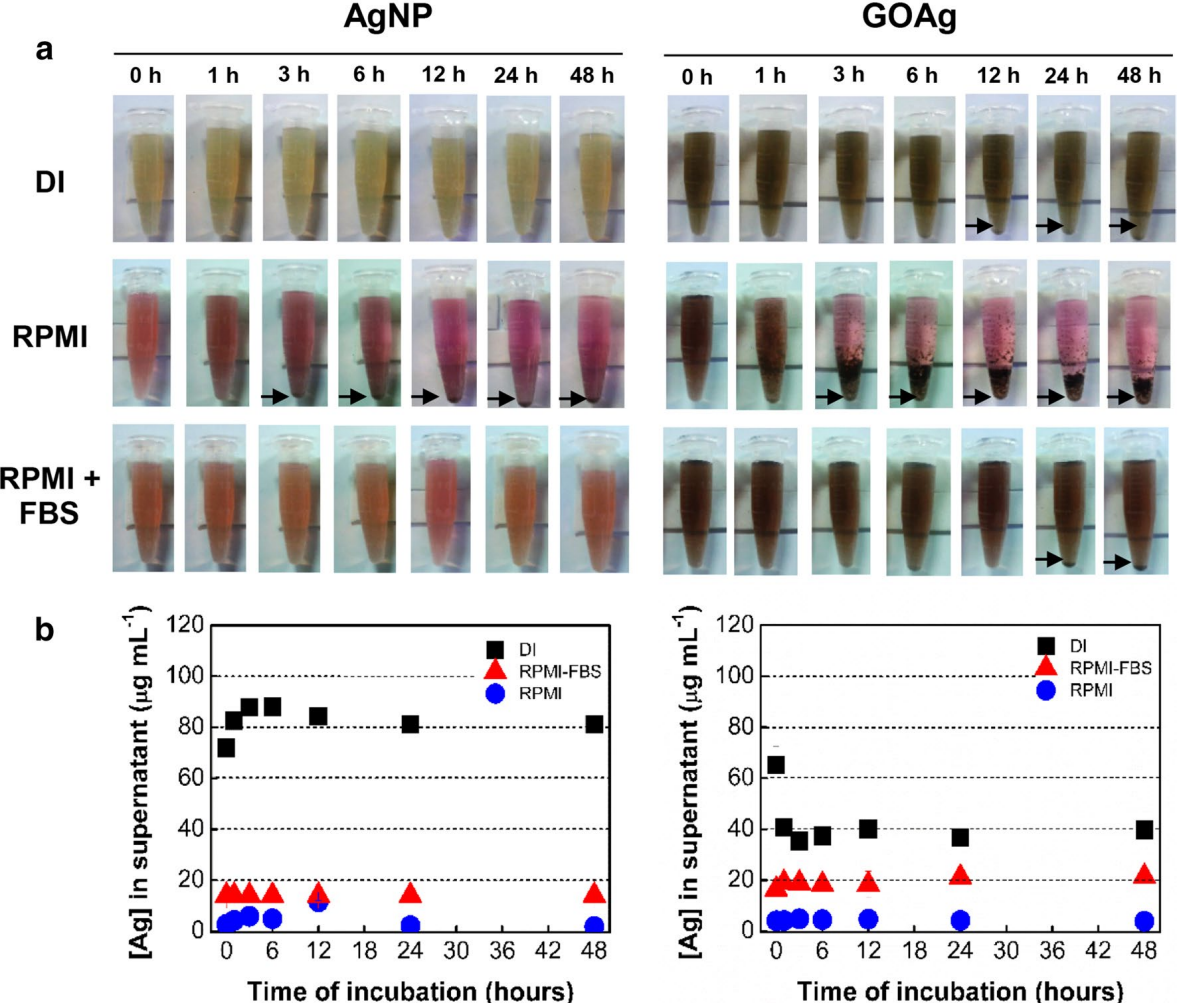
Stability of pristine silver nanoparticles and graphene oxide-silver nanocomposites in cell culture media. **a** Photographs of nanomaterials dispersed in DI water, RPMI, and RPMI + 10 % FBS over 48 h of incubation. *Black arrows* indicate precipitation of nanomaterials. **b** Concentration of silver present in the supernatant of centrifuged pristine silver nanoparticles and graphene oxide-silver nanocomposite previously dispersed in DI water and cell media. Initial concentration of Ag = 80 µg mL^−1^

Because the stability of nanomaterials in physiological media is an important feature to further biomedical applications, the stability of pristine AgNP and GOAg nanocomposite was investigated after dispersion in DI water and RPMI cell culture medium that was either supplemented or not with fetal bovine serum (FBS). The nanomaterials’ stability was estimated by visual observation (Fig. 2a) and through determining the concentration of silver in the supernatant (Fig. 2b).

The pristine AgNP did not visually display any precipitation when the nanoparticles were dispersed in DI water, regardless of the incubation time (Fig. 2a). However, the GOAg nanocomposite exhibited precipitation in DI water after 12 h of incubation (Fig. 2a). Both nanomaterials showed strong precipitation after 3 h of incubation when they were dispersed in RPMI medium without FBS. In contrast, the RPMI medium supplemented with 10 % FBS maintained the pristine AgNP highly stable over 48 h of incubation. However, the GOAg nanocomposite underwent precipitation only after 24 h of incubation. The stability of pristine GO is available in the supplementary material (Additional file 3: Figure S3). The GO partially precipitated in both DI water and RPMI medium supplemented with 10 % FBS after 6 h of incubation, whereas strong precipitation of this nanomaterial was observed in RPMI medium in the same period (Additional file 3: Figure S3).

Figure 2b highlights the concentration of silver in the supernatants of pristine AgNP and GOAg nanocomposite dispersed in DI water and RPMI medium (supplemented or not with 10 % FBS). Regardless of the dispersion medium, the initial concentration of silver for both samples of the nanomaterials was 80 µg mL^−1^. The stability of pristine AgNP was considerably reduced when the nanoparticles were dispersed and centrifuged in cell media. For instance, the silver concentrations in the supernatants of pristine AgNP dispersed in RPMI and RPMI supplemented with FBS were reduced to 2.7 and 14 µg mL^−1^, respectively, regardless of the incubation period (Fig. 2b). In contrast, the concentration of silver in the supernatant of pristine AgNP dispersed in DI water remained similar to the initial concentration over the 48 h of incubation, demonstrating its high stability in aqueous medium.

No variation in the silver concentration was observed in the GOAg supernatant immediately after dispersion and centrifugation in DI water (Fig. 2b). Therefore, the concentration of silver in the supernatant decreased to 40 μg mL^−1^ after 1 h and this value remained constant over a period of 48 h (Fig. 2b). In contrast, the silver concentration in the supernatant of GOAg dispersed in RPMI and RPMI supplemented with FBS media was reduced to 3.8 and 21.4 μg mL^−1^, respectively, without significant variation over 48 h.

The agglomeration state of pristine GO, pristine AgNP, and GOAg nanocomposite in DI water and cell media was determined by dynamic light scattering (DLS). The hydrodynamic sizes are shown in the supplementary information (Additional file 4: Table S1). The hydrodynamic sizes of GO aggregates decreased in the following order: 10,000 nm in RPMI > 5956 nm in DI water >4600 nm in RPMI supplemented with FBS. Regardless of the dispersion medium, the pristine GO hydrodynamic sizes diminished progressively over 48 h, probably owing to the early precipitation of the larger aggregates.

The hydrodynamic sizes of pristine AgNP dispersed in DI water slightly increased from 22.8 to 29.3 nm after 48 h of incubation. Pristine AgNP dispersed in RPMI medium exhibited hydrodynamic sizes >1000 nm, suggesting strong agglomeration over the period of incubation. However, when pristine AgNP was dispersed in RPMI supplemented with FBS, the hydrodynamic sizes slightly increased from 33.7 to 35.6 nm after 48 h. It is important to mention that the presence of the FBS reduced the polydispersity index from 0.5 to 0.2, suggesting that the nanoparticles became more monodispersed in the cell medium supplemented with FBS.

The GOAg nanocomposite hydrodynamic sizes in DI water decreased from 222.1 to 152.5 nm after 48 h. Moreover, when GOAg was dispersed in RPMI medium, its hydrodynamic size increased to 2385 nm and the aggregates increased to 4647 nm after 24 h. The dispersion of GOAg in the RPMI supplemented with FBS reduced the aggregation state over 48 h compared to the RPMI alone. The hydrodynamic sizes of the GOAg aggregates in RPMI supplemented with FBS slightly increased from 197.9 to 231.4 and 218.1 nm, after 24 and 48 h, respectively. The polydispersity index varied between 0.3 and 0.4, similar to that obtained when GOAg was dispersed in DI water.

The nanomaterials presented greater stability in DI water; however, GOAg was less stable in the cell media than in the pristine AgNP. For example, the concentration of silver in the GOAg supernatant was reduced by almost 50 % after 1 h incubation, while the silver concentration in the pristine AgNP supernatant did not vary under identical experimental conditions. The oxygenated functional groups on the GO surface maintain the stability of the nanomaterial in an aqueous medium while providing nucleation sites for the growth and stabilization of the silver nanoparticles [37, 38]. However, centrifugation contributed to the nanocomposite agglomeration and precipitation, thus decreasing the concentration of silver in the supernatant.

Concerning the cell culture media, the nanomaterials were more stable when dispersed in RPMI supplemented with FBS than in the medium without serum. RPMI medium has a high ionic strength due to the presence of numerous components containing chloride ions. These ions are well-known aggregating agents for silver nanoparticles, which may lead to the formation of AgCl complexes, a crystalline solid with low solubility in water [39, 40]. It is worth noting the presence of cysteine in the RPMI media. This amino acid promptly adsorbs onto the AgNP surface and reduces its stability in aqueous media because of the high energy of the –SH bonds in the amino acid structure [40, 41].

### Cytotoxicity of the nanomaterials

**Fig. 3:**
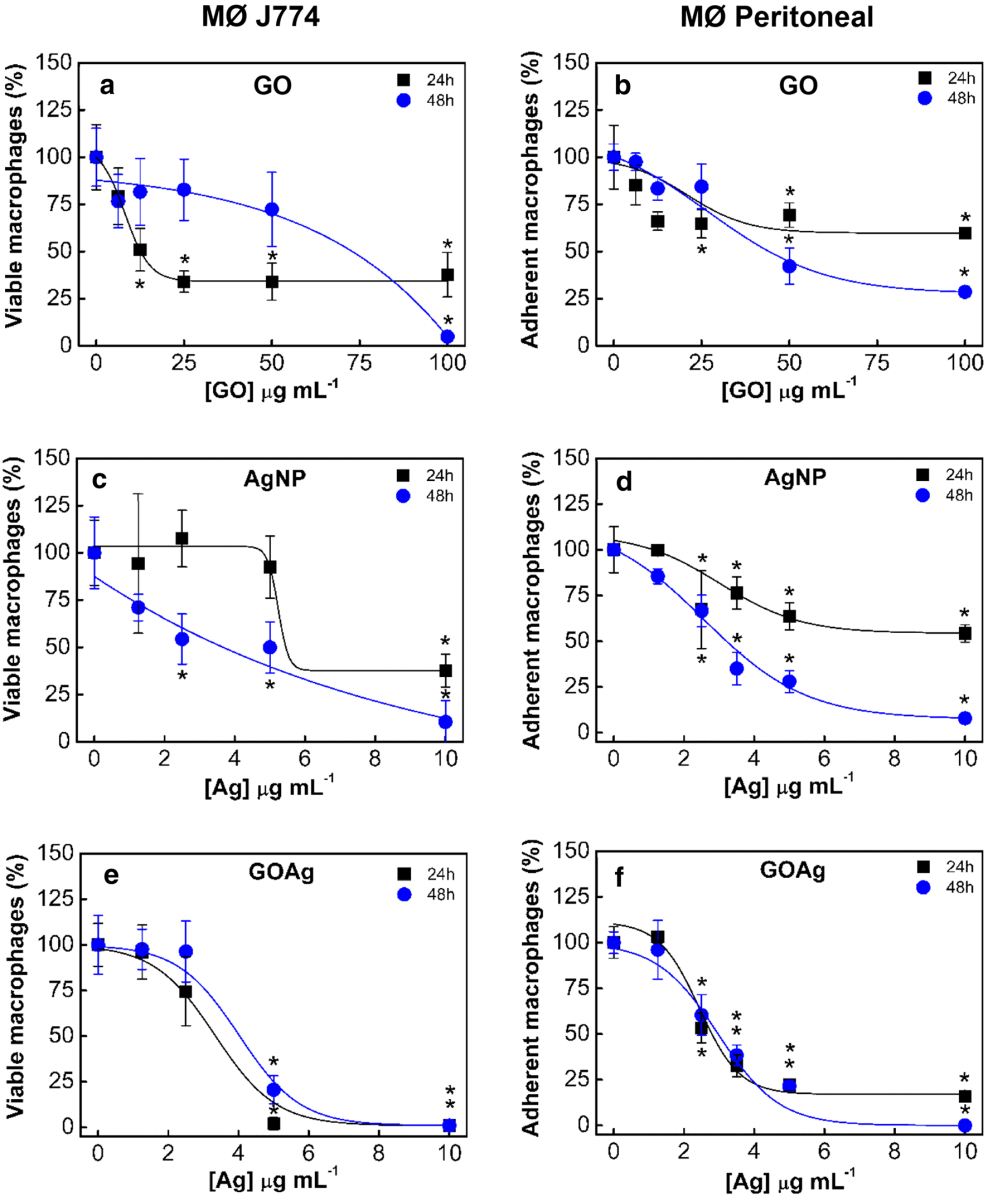
Cytotoxicity of nanomaterials to J774 tumoral macrophages and peritoneal macrophages from Balb/c mouse. The number of viable tumoral macrophages was determined by trypan blue exclusion assay. The percentage of peritoneal macrophages adhered to the coverslips was determined after exposure to nanomaterials. Pristine graphene oxide (**a**, **b**), pristine silver nanoparticles (**c**, **d**), and graphene oxide-silver nanocomposite (**e**, **f**) after 24 and 48 h of exposure. Differences in the cytotoxicity of nanomaterial concentrations were determined compared to control cells (*) p < 0.05. *MØ* macrophage

The cytotoxicity of the nanomaterials occurred in a dose–response manner and the results are reported as the percentage of viable cells (Fig. 3). The IC_50_ values for the J774 and peritoneal macrophages are present in Table 2. Interestingly, pristine GO was more toxic to J774 macrophages after 24 h (IC_50–24 h_ = 16.9 µg mL^−1^) than after 48 h (IC_50–48 h_ = 58.4 µg mL^−1^) of exposure (Table 2). This result indicates that the tumoral cells that survived for 24 h proliferated. In contrast, pristine GO was more toxic to peritoneal cells after 48 h of exposure than after 24 h, with IC_50–24 h_ = 32.9 and IC_50–48 h_ = 24.7 µg mL^−1^, respectively (Table 2). In contrast to immortalized tumoral cells, primary macrophages are bone marrow-derived cells and do not reproduce by themselves [30], thus negating any possibility of cell culture resilience attributed to proliferation.

**Table 2:** Summary of the toxicity of graphene-silver nanocomposite and its counterparts toward macrophages

Nanomaterial (µg mL^−1^)	IC_50–24 h_	CI 95 %	IC_50–48 h_	CI 95 %
MØ J774				
GO	16.9	(10.1–28.2)	58.4	(42.4–80.5)
AgNP	8.9	(7.0–11.3)	2.9	(1.8–4.6)
GOAg	2.9	ND	3.8	ND
MØ peritoneal				
GO	32.9	ND	24.7	ND
AgNP	10.4	(7.2–14.9)	3.0	(2.4–3.75)
GOAg	3.1	(2.5–3.8)	2.9	(2.0–4.2)

*IC50* 50 % inhibitory concentration,* CI 95 %* confidence interval,* MØ* macrophage,* ND* not determined. The IC50 and CI are expressed in µg mL^−1^

For instance, Mendes et al. reported that after testing the GO cytotoxicity (10 µg mL^−1^) using the trypan blue assay, the viability of J774.2 macrophages was not reduced after 48 h [16]. Yue et al. demonstrated that GO concentrations up to 20 µg mL^−1^ induced no relevant viability reduction to primary macrophages isolated from mouse and J774A.1 tumoral macrophages after 48 h [15]. Although neither study addressed the IC_50_ values, our findings reveal that concentrations of GO below 12.5 µg mL^−1^ did not significantly reduce the viability of J774 macrophages (Fig. 3a) after 48 h, which are in agreement with Mendes et al. and Yue et al. Moreover, no significant viability reduction of peritoneal macrophages was observed for GO at concentrations below 25 µg mL^−1^, regardless of the exposure time (Fig. 3b). Despite both authors have not addressed the viability reduction at higher concentrations of GO, our data shows markedly increases of GO toxicity up to 100 µg mL^−1^ for tumoral and peritoneal macrophages. The toxicity of graphene to macrophages relies on the capacity of those cells to internalize these nanomaterials leading to inflammation, increased mitochondrial respiration rate and apoptosis [42]. However, graphene-based materials may damage the cell membrane and exhibit toxicity without being necessarily internalized [12].

Pristine AgNP exhibited dose- and time-dependent cytotoxicity for both macrophages (Fig. 3c, d). The toxicity toward J774 macrophages increased approximately threefold from 24 h (IC_50–24 h_ = 8.9 µg mL^−1^) to 48 h (IC_50–48 h_ = 2.9 μg mL^−1^) of exposure (Table 2). Identical phenomena were observed for peritoneal macrophages from 24 h (IC_50–24 h_ = 10.4 µg mL^−1^) to 48 h (IC_50–48 h_ = 3.0 µg mL^−1^) of exposure (Table 2). Therefore, the IC_50_ values indicate that AgNP toxicity to both macrophages is similar and these nanoparticles are more toxic than pristine GO (Table 2).

Pratsinis et al. [43] studied the toxicity of silver nanoparticles synthesized without any organic surface coating and with a well-defined size distribution (5.7–20.4 nm) to RAW 264.7 murine macrophages. Although the authors used a different macrophage lineage, our toxicity findings for pristine AgNP with a size distribution from 5.1 to 19.1 nm were similar to those of Pratsinis et al., who obtained IC_50_ values between 7 and 20 µg mL^−1^ for the same exposure periods.

Zhang et al. compared the toxicity of citrate-stabilized silver nanoparticles of 22 ± 3.5 nm to macrophages of different lineages: THP-1 macrophages from human peripheral blood and RAW 264.7 murine macrophages. The toxicity toward both macrophages was similar. A silver concentration of 50 µg mL^−1^ reduced the viability of the macrophages by approximately 20 % after 24 h of exposure. Our results for pristine AgNP indicate that a much smaller concentration of silver (5 μg mL^−1^) was sufficient to cause a similar reduction in macrophage viability at the same exposure time (Fig. 3c, d). These divergences in toxicity may be related to the physicochemical characteristics of the nanoparticles, such as size, shape, surface charge, surface coating, and stability. The lineage of the macrophages must also be considered [44].

GOAg cytotoxicity occurred in a dose-dependent manner for both macrophages and did not increase over time (Fig. 3e, f). The GOAg IC_50_ for J774 macrophages were 2.9 and 3.8 µg mL^−1^ after 24 and 48 h of exposure, respectively. GOAg presented a very similar cytotoxicity toward peritoneal macrophages, with IC_50_ values of 3.1 and 2.9 µg mL^−1^ after 24 and 48 h of exposure, respectively (Table 2).

Data about the toxicity of graphene-metal nanocomposites toward macrophage cells are scarce in the literature. However, Zhou et al. reported that a graphene oxide/silver nanocomposite with a mass ratio of 20:1 (GO:Ag) reduced the viability of both A549 lung tumor epithelial cells and HepG2 hepatocellular carcinoma cells to 64.2 and 60 % respectively, after 24 h exposure and it was significantly more toxic than equivalent concentrations of bare AgNP [45].

In the present work, the GOAg nanocomposite with an equivalent mass ratio of GO:Ag equal to 1:1 (Table 1) was tested at non-toxic concentrations of GO (below 10 μg mL^−1^) (Fig. 3a, b); and it reduced the viability of both macrophages to 25 % after 24 h of exposure (Fig. 3e and F). Moreover, GOAg was found to be more toxic than its pristine counterparts to the macrophages.

The toxicity of GOAg toward bacterial cells is reported in the literature to be a result of a synergistic effect between GO and AgNP. Xu et al. described this effect as a “capture-killing process” capable of inhibiting the bacterial growth due to graphene adsorption properties and silver bactericidal activity [10]. De Faria et al. [15] and de Moraes et al. [46] also reported a similar synergistic effect of GOAg nanocomposite toward bacterial cells. These authors attributed this effect to the large surface area of GO sheets, which enables bacterial attachment and allows intimate contact between the AgNP and the surface of the cells. The toxicity mechanisms associated with the synergistic effect of GOAg nanocomposites toward cells may include disruption of cell wall/membrane integrity and inhibition of cell division [47], and generation of oxidative stress [48]. Therefore, the toxicity of GOAg nanocomposite toward macrophage cells may also be related to the synergistic effect, which maximizes the contact between the cells and the AgNP attached to GO.

### Oxidative stress

**Fig. 4:**
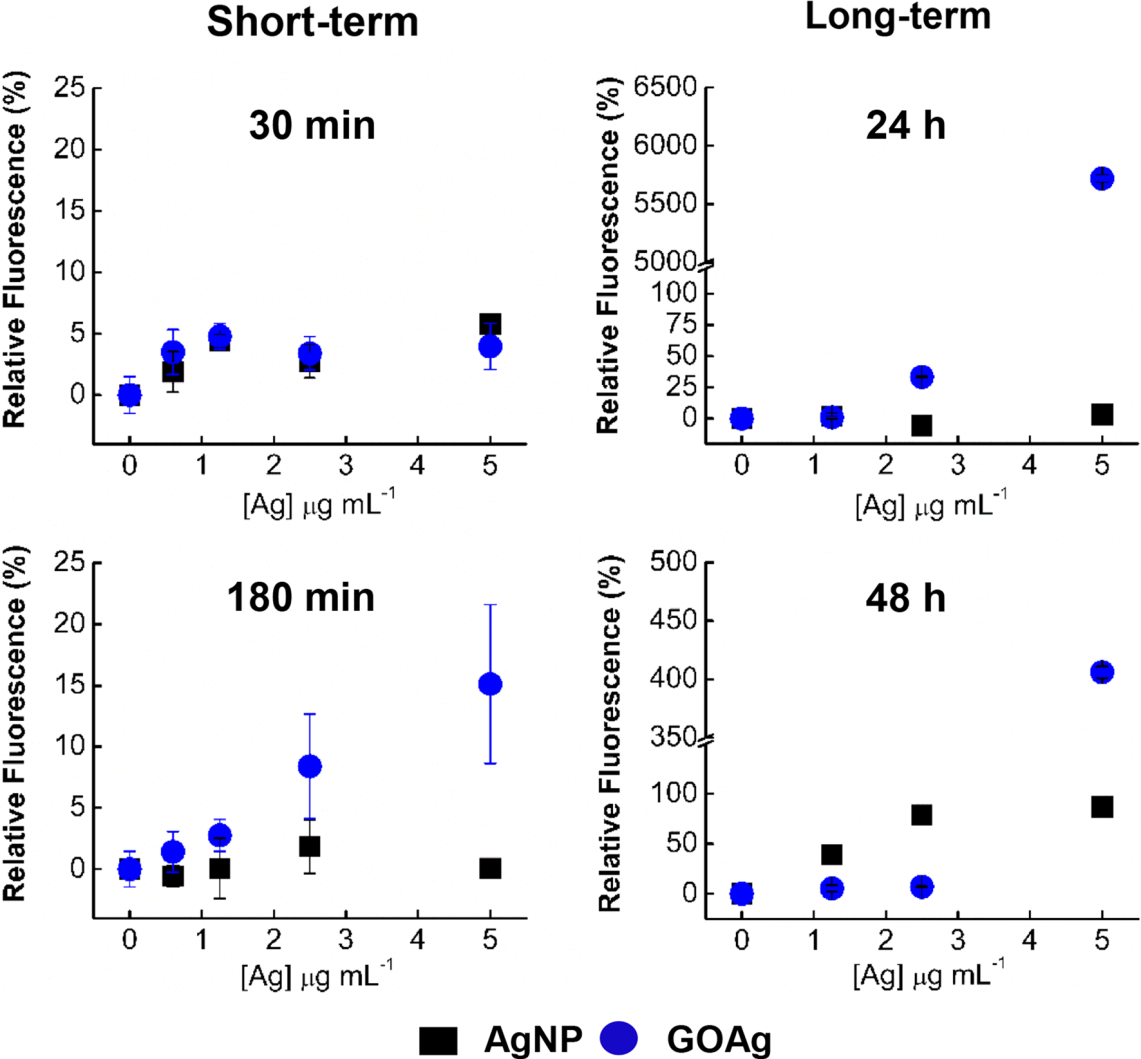
Production of reactive oxygen species after exposure of J774 tumoral macrophages to the nanomaterials. Percentage of fluorescence relative to control (cells without the presence of nanomaterials) after exposure for 30 and 180 min in the short-term assessment, as well as after 24 and 48 h in the long-term assessment. The fluorescence was normalized according to the number of viable cells

Cells naturally produce intracellular reactive oxygen species (ROS) by the mitochondrial electron transport chain; however, antioxidant enzymes promptly detoxify these free radicals [49]. Nonetheless, when impairment of mitochondrial function occurs, such as the reduction of the membrane potential, excess superoxide radicals are produced, thus causing oxidative stress [50, 51]. Macrophages produce free radical species in response to invaders such as intracellular parasites [52], viruses [53], and other potential stressors, such as nanomaterials [54]. We therefore compared the capacity of pristine AgNP and GOAg to induce ROS production in J774 tumoral macrophages (Fig. 4). Because those radicals are frequently produced during exposure to the stressor [55], we quantified ROS production after short-term (30 and 180 min) and long-term (24 and 48 h) exposure to the nanomaterials.

No relevant fluorescence was observed after 30 min of exposure to both nanomaterials. However, after 180 min, GOAg significantly induced the macrophage to produce ROS, increasing the fluorescence in a dose-dependent manner (Fig. 4). Regarding long-term exposure, the fluorescence of cells exposed to GOAg increased almost 60-fold after 24 h. On the other hand, the relative fluorescence of macrophages exposed to GOAg was significantly reduced after 48 h, probably due to the reduction in the production of superoxide radicals (Fig. 4). Pristine AgNP induced macrophages to produce significant ROS only after 48 h, when the macrophages fluorescence doubled after exposure to 2.5 and 5 µg mL^−1^ of silver.

The fluorescence of cells exposed to GO alone was also determined (data not shown). Negative values of relative fluorescence were found after macrophage exposure to GO alone at concentrations up to 50 µg mL^−1^, possibly owing to fluorescence quenching [22]. Although we could not determine the ROS production induced by GO alone, this nanomaterial may also contribute to ROS generation. Qu et al., [23] reported oxidative stress in J774A and RAW 264.7 macrophages induced by GO. These authors found that fluorescence of both cells increased progressively and in a dose-dependent manner. The cells produced an approximately 1.8-fold fluorescence after 10 min of exposure to 10 μg mL^−1^ of GO.

Therefore, pristine AgNP and GOAg nanocomposite induced a dose- and time-dependent ROS production in macrophages; the nanocomposite also induced an earlier oxidative stress. The biological effects of metallic nanoparticles, such as oxidative stress, may be attributed to the “Trojan horse effect”, a phenomenon observed when internalized nanoparticles are subjected to the metabolic and digestive intracellular pathways, provoking the release of metal ions that induce ROS production and other toxic outcomes [56–58].

Recently, Gurunathan et al. reported that the synergistic toxicity effect of reduced graphene oxide/silver nanoparticles nanocomposite to epithelial ovarian carcinoma cells (A2780) is related to membrane stress and oxidative stress [59]. In the present study, and in accordance with the cell viability data (Fig. 3), toxic concentrations of GOAg induced more oxidative stress than did the pristine AgNP, which may be a result of a synergistic oxidative stress of the nanocomposite toward the macrophages.

### Nanomaterials: cell interaction and uptake by macrophage

**Fig. 5:**
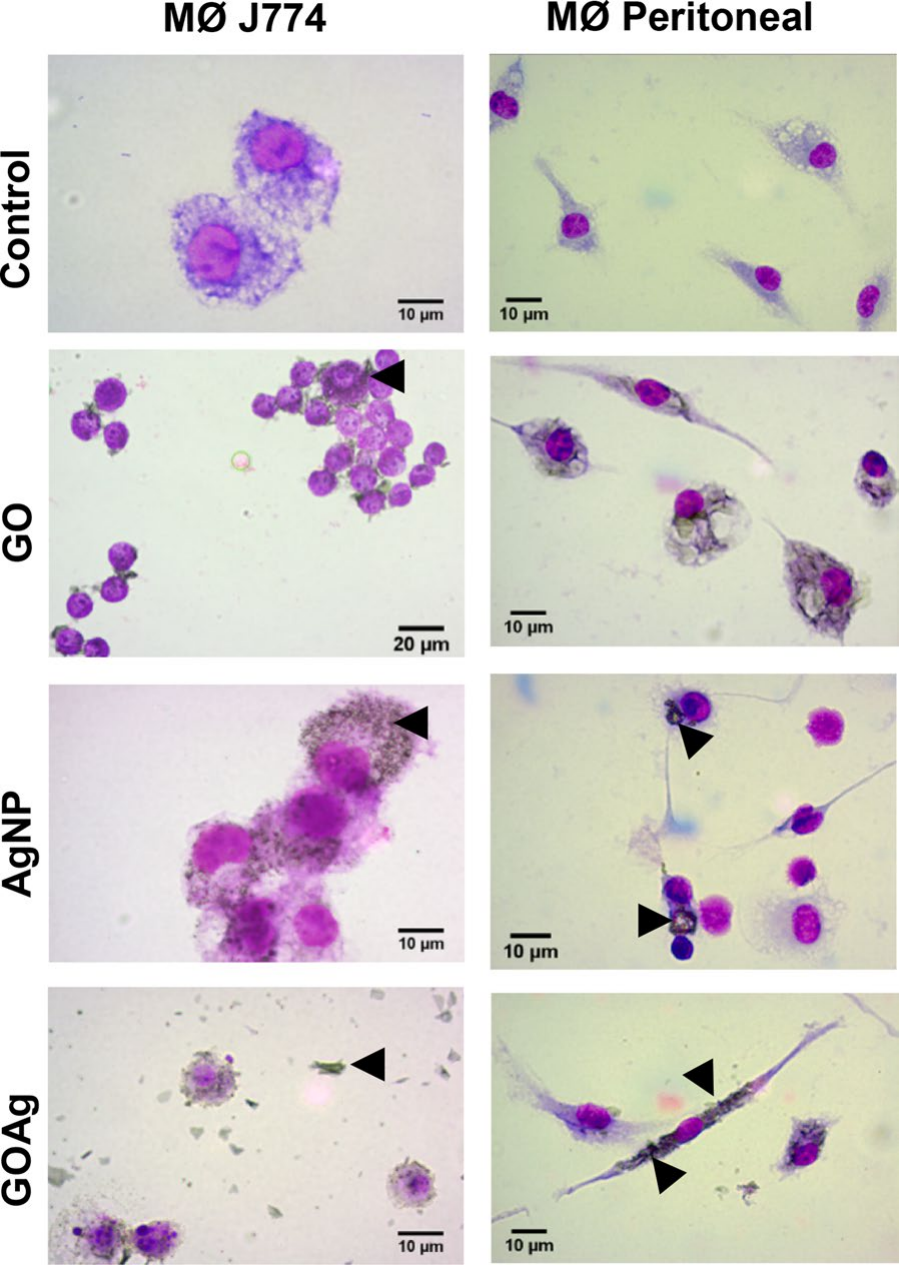
Light micrographs of tumoral J774 and peritoneal macrophages. Cells were stained with Giemsa after 48 h exposure. (Control) Cells cultivated in microplates without nanomaterials. (GO) Cells exposed to 12.5 μg mL^−1^ pristine graphene oxide. *Black arrows* indicate the interaction between the graphene oxide sheets and macrophage surface. (AgNP) cells after exposure to 5 μg mL^−1^ silver nanoparticles. *Black arrows* highlight silver nanoparticles internalization. (GOAg) cells after exposure to 5 μg mL^−1^ graphene oxide-silver nanocomposite. *Black arrows* indicate nanocomposite flakes in cell media and interaction with cellular surface. *MØ* macrophage

The interaction of the nanomaterials with J774 and peritoneal macrophages was primarily studied by optical microscopy (Fig. 5). In general, the macrophages exposed to pristine GO, pristine AgNP and GOAg nanocomposite did not exhibit morphological changes compared to the control cells. However, large and small vacuoles were observed in both macrophages exposed to the nanomaterials. Micrometric agglomerates of GO and GOAg were found interacting with cells of both macrophages as shown by the arrowheads (Fig. 5).

**Fig. 6:**
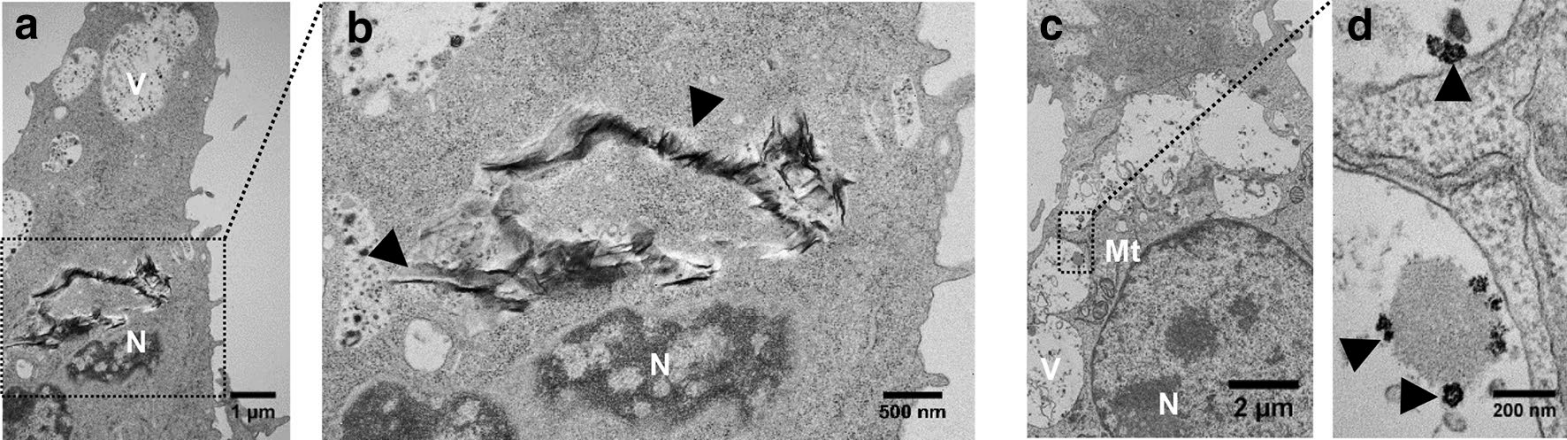
Internalization of pristine graphene oxide and pristine silver nanoparticles by J774 tumoral macrophage. **a** Cells with internalized pristine graphene oxide (13,000×); *black arrowheads* indicate pristine graphene oxide agglomerates localized close to the nucleus (**b**) (22,000×). **c** and **d** highlight the internalization of pristine silver nanoparticles (10,000×), in the detail, *black arrowheads* point to agglomerated nanoparticles localized in the vacuoles. *N* nucleus, *V* vacuole, *Mt* mitochondria

Further investigation was performed by TEM analysis of the J774 macrophages. Pristine GO was internalized by the J774 macrophage cells (Fig. 6a, b) as previously observed by other authors [16, 18]. Pristine AgNP was also internalized by the J774 cells (Fig. 6c, d) and strong agglomeration was evident inside macrophage vacuoles. Similar agglomeration of silver nanoparticles was observed in multilamellar bodies of dendritic cells and lung epithelial cells (A549) [60]. The AgNP agglomerates inside the cells reached approximately 100 nm (Fig. 6d), indicating that aggregation increased the size of the nanoparticles approximately eightfold compared to the pristine AgNP (Fig. 1d).

**Fig. 7:**
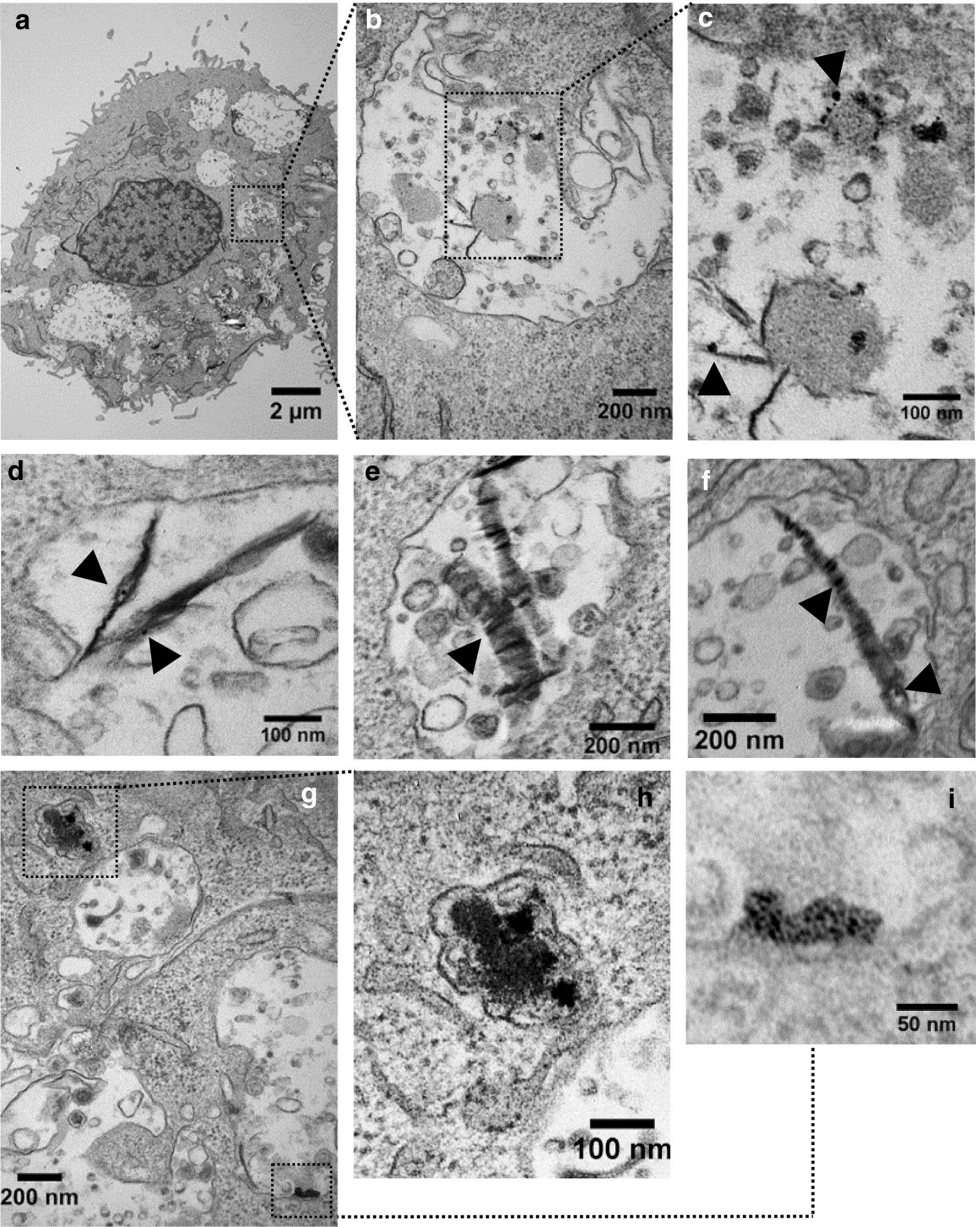
Internalization of graphene oxide-silver nanocomposite by J774 tumoral macrophages. **a** Macrophage in low magnification (6000×). **b** Detail of vacuole containing the internalized nanocomposite. **c** Highlight of the nanocomposite inside the vacuole with *black arrowheads* pointing to silver nanoparticles on graphene oxide sheets. **d**, **e**, and **f** amplified images of the nanocomposite inside cell vacuoles. The *black arrowheads* indicate silver nanoparticles on graphene oxide sheets. **g** High-magnification of cell compartments containing degraded GOAg nanocomposite (47,000×). **h** Detail of the cell compartment containing degraded GOAg nanocomposite and close to a vacuole. **i** Detail of a partially degraded GOAg sheet and close to the border of a vacuole

The GOAg was also successfully internalized by the J774 macrophages, as shown in images of the vacuoles containing the nanocomposite (Fig. 7a–i). Figure 7b–g shows in detail the internalized nanocomposite. The arrowheads highlight the AgNP attached to GO sheets inside the vacuoles. Moreover, the TEM images (Fig. 7g–i) display the macrophage compartments containing partially degraded GOAg. The degradation of the nanocomposite may be a result of the enzymatic activity, which can cleave the carbon–carbon bonds and form holes in the graphene basal plane [61]. In vitro studies demonstrated the effective biodegradation of carbonaceous nanomaterials by enzymes derived from leukocyte cells [32, 62–64]. In addition, metallic nanoparticles, such as AgNP, dissolve in acidic pH and can be rapidly degraded after entering the endosomal pathway [40].

**Fig. 8:**
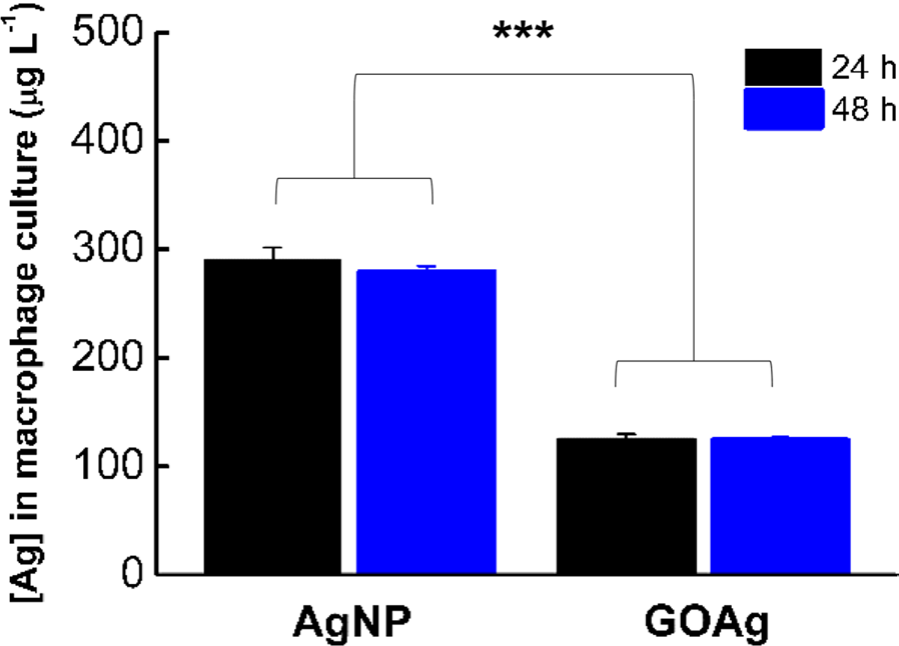
Concentration of silver inside J774 tumoral macrophage. Cells cultivated in culture bottles exposed to 1000 µg L^−1^ of pristine silver nanoparticles and graphene oxide-silver nanocomposite for 24 and 48 h. Nanomaterial internalization by cells was estimated by measuring silver concentration ([Ag]) using ICP-MS. (***) p < 0.001. The [Ag] was normalized by the number of cells

The uptake of silver by J774 tumoral macrophages was determined by ICP-MS after 24 and 48 h incubation with pristine AgNP and GOAg nanocomposite (Fig. 8). The initial concentration of silver in the experiment was the same for both nanomaterials (1000 µg L^−1^). Macrophages internalized approximately 30 % of the initial concentration of silver when exposed to pristine AgNP (291.2 and 278.9 µg L^−1^ of silver after 24 and 48 h, respectively). However, when the cells were exposed to GOAg, they internalized only 12 % of silver (124 and 124.2 µg L^−1^ of silver after 24 and 48 h, respectively).

The exposure of the cells to the nanomaterials for 24 h was sufficient for the macrophages to internalize both the pristine AgNP and GOAg nanocomposite, and no significant variation was found in the concentration of internalized silver during the additional 24 h of incubation. However, the GOAg was barely internalized by the macrophages. This finding may be explained by the morphological features of this nanocomposite. The lateral dimensions and size of the bidimensional sheets may difficult/reduce the uptake by cells compared to pristine AgNP, which are much smaller and have a spherical morphology [65, 66]. Additionally, the GO can strongly interact with the plasma membrane [18, 67] and hinder the internalization process.

Zhou et al. showed that epithelial cells from human lung carcinoma (A549) internalized more silver after exposure to graphene oxide/silver nanocomposite compared to the pristine silver nanoparticles [45]. As previously noted, we observed a reduction in silver internalization when macrophages were exposed to the GOAg nanocomposite. Our findings might be attributed to several causes, such as different cellular lineages, the physicochemical characteristics of the nanomaterials, and mainly to the fact that we tested non-toxic concentrations of nanomaterials, to avoid the reduction of the number of cells, and consequently of the uptake level.

**Fig. 9:**
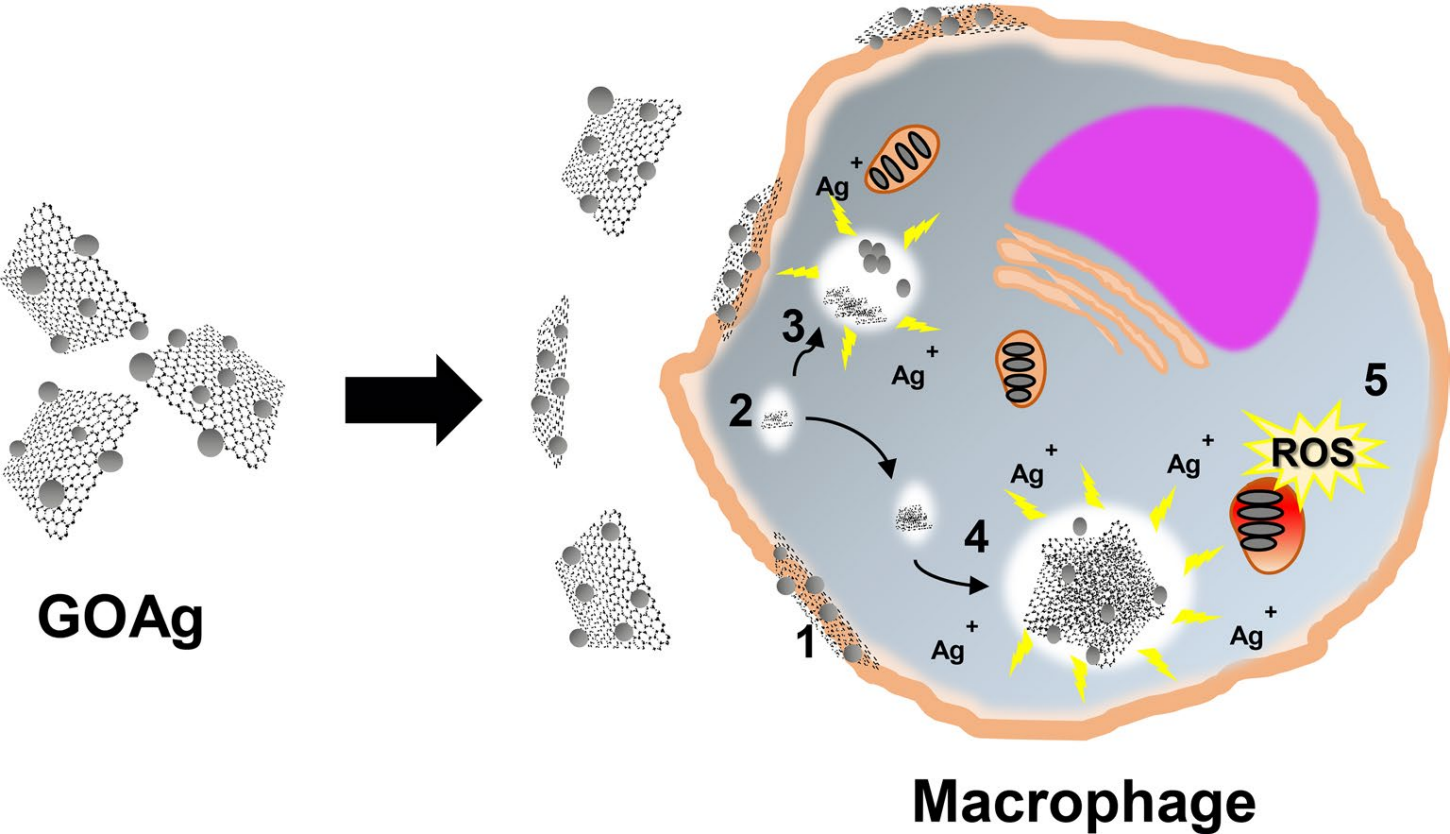
Scheme of the uptake and degradation of the nanocomposite and generation of oxidative stress by the macrophage cells. The scheme represents the general steps of the graphene oxide-silver nanoparticles and cell interaction, including the nanocomposite interaction with the cell membrane (*1*), macrophage endocytosis and vesicle maturation (*2*), nanocomposite degradation (*3*), release of silver ions in the cytoplasm (*4*), and impairment of mitochondrial function generating toxic radicals and oxidative stress (*5*)

The schematic representation of the basic mechanism of the GOAg-macrophage interaction is shown in Fig. 9. The GOAg nanocomposite interacts with the macrophage cell membrane, leading to endocytic internalization. The nanocomposite ends up in vacuoles where degradation can take place. Thus, the combination of the degradation of GOAg and the release of silver ions from the AgNP to the cytoplasm can disrupt mitochondrial activity, causing oxidative stress and macrophage death.

In summary, this study compared the cytotoxicity of GOAg nanocomposite and its counterparts toward macrophages. The reduction of cell viability and the increased oxidative stress induced by the GOAg nanocomposite suggest a synergistic effect. To the best of our knowledge, this is the first report concerning the cytotoxicity of graphene oxide-silver nanocomposite and its counterparts toward macrophages that also investigated the nanomaterials’ stability in different media and their internalization. Despite the novelty of this work, additional work is still needed to better comprehend the cytotoxicity mechanisms caused by graphene-metal based nanocomposites.

## Conclusions

In this work, we assessed the toxicity of graphene oxide-silver nanocomposite and its pristine counterparts toward macrophages from a tumoral lineage and collected from mouse peritoneum. We also investigated the nanomaterials stability in cell media. The nanocomposite was found to be more toxic than pristine GO and pristine AgNP to both macrophages, without significant variations between the lineages. Moreover, GOAg nanocomposite exhibited earlier effects to all the evaluated endpoints (cell viability and oxidative stress). Its toxicity may be a result of a synergistic effect between the GO sheets and the AgNP, because GOAg sheets can maximize the contact between silver nanoparticles and cells. TEM images revealed that GOAg was successfully internalized by the tumoral macrophages and showed that the nanocomposite can be degraded by the cell. However, the ICP-MS analysis showed that GOAg was less internalized by the tumoral cell compared to pristine AgNP. The nanomaterials were poorly stable in the cell media, which concerns future in vivo applications. The toxicity and fate of nanomaterials in cells are some of the major concerns in the development of biocompatible materials and must be carefully evaluated.

## Methods

### Synthesis of graphene oxide (GO)

Graphene oxide sheets were synthesized by the modified Hummers method [4, 36]. Natural graphite (Synth, Brazil) was pretreated to ensure complete oxidation. For this, graphite powder (1.0 g) was heated (90 °C) in concentrated sulfuric acid (H_2_SO_4_, 4.4 mL) containing potassium persulfate (K_2_S_2_O_8_, 0.8 g) and phosphorus pentoxide (P_2_O_5_, 0.8 g). The acidic mixture was kept stirring on a hotplate for 4.5 h. Shortly afterward, the mixture was diluted in DI water and allowed to stand overnight. Next, it was filtered using a 0.22 μm PVDF membrane (Millipore), washed with DI water, and the isolated solid was dried at room temperature overnight.

For the oxidation step, the pretreated graphite was added to a chilled flask (0 °C) containing H_2_SO_4_ (40 mL). Potassium permanganate (KMnO_4_, 5.0 g) was gradually added to the mixture and the temperature was controlled to avoid exceeding 10 °C. The ice bath was removed and the mixture was allowed to react at 35 °C for 2 h. Then DI water was added in small aliquots using an ice bath to maintain the temperature below 50 °C. The mixture was kept stirring for an additional 2 h. Immediately afterward, more DI water was added to the mixture, followed by the addition of hydrogen peroxide (H_2_O_2_, 30 % v v^−1^). The resulting bright-yellow mixture was left to settle for 2 days. The mixture was filtered using a 0.22 μm PVDF membrane (Millipore), followed by centrifugation with hydrochloric acid (HCl, 10 % v v^−1^) and DI water to remove metal ions and acid, respectively. The resulting product was dialyzed (Fisherbrand dialysis tubing 12,000–14,000 Da) against DI water for 10 days to remove residual salts. The graphene oxide dispersion was lyophilized and stored at room temperature in screw-cap propylene tubes protected from humidity and light.

### Synthesis of pristine silver nanoparticles (AgNP)

Pristine silver nanoparticles were synthesized by the Turkevich method [68]. Silver nitrate (AgNO_3_, 8.4 mg) was dissolved in 40 mL of DI water and heated at reflux. As soon as the solution began to boil, a sodium citrate solution (10 mL, 1 mmol L^−1^) was added dropwise. The reaction was kept between 110 and 130 °C for 30 min. The AgNP dispersion was dialyzed against DI water for 48 h to remove residual salts (Fisherbrand dialysis tubing 12,000–14,000 Da) and stored in a chilled vessel protected from light.

### Synthesis of graphene oxide-silver nanocomposite (GOAg)

The GOAg was synthesized by the modified Turkevich method [13, 46]. For this, GO (6.2 mg) was dispersed in DI water (20 mL) and sonicated for 30 min. Next, AgNO_3_ (8.4 mg) dissolved in DI water (20 mL) was added to the previous GO dispersion and submitted to sonication for an additional 30 min. The mixture was heated at reflux, and when the temperature reached approximately 110 °C, a sodium citrate solution (10 mL, 1 mmol L^−1^) was added dropwise. The reaction was kept at 130 °C for 50 min. The GOAg dispersion was dialyzed for 48 h to remove residual salts (Fisherbrand dialysis tubing 12,000–14,000 Da) and stored in a sealed vessel protected from light.

### Characterization of nanomaterials

The formation of AgNP was confirmed by the plasmon resonance band using ultraviolet–visible spectroscopy (UV–Vis, Shimadzu UV-1650 PC spectrometer). The morphology of GO, AgNP, and GOAg was observed by transmission electron microscopy (TEM, Zeiss Libra 120, accelerating voltage of 120 kV). The thickness of the GO sheets was determined by atomic force microscopy (AFM, Shimadzu SPM-9600). X-ray diffraction was performed using a Shimadzu XRD-7000 diffractometer. The size distribution of the AgNPs was calculated after counting approximately 600 particles in several TEM images using the Image J software. The hydrodynamic size and the zeta potential were performed using a Zeta potential analyzer (Zetasizer Nano ZS, Malvern). The concentration of silver in the AgNP and GOAg dispersions was quantified by optical emission spectrometry with inductively coupled plasma (ICP OES, Perkin Elmer, OPTIMA 8300 DV). Before the ICP analysis, the samples were digested in concentrated nitric acid (HNO_3_, Synth) for 24 h and suspended in ultrapure water.

### Stability of GO, AgNP, and GOAg in cell culture media

The stability of the nanomaterials was investigated in cell culture media. Briefly, a stock dispersion of pristine AgNP was diluted in RPMI 1640 medium (Nutricell) supplemented with fetal bovine serum (10 % FBS, Nutricell) to reach a silver concentration of 80 µg mL^−1^. The samples were then incubated for 1, 3, 6, 12, 24, and 48 h in a cell culture chamber at 37 °C with 5 % CO_2_ and pH 7.4. Shortly after each incubation period, the samples were centrifuged at 14,000 rpm for 30 min (Eppendorf 5804R). An aliquot of the supernatant (1 mL) was carefully withdrawn, digested in HNO_3_, and the total amount of silver (Ag^0^ and Ag^+^) was determined by ICP OES. The same procedure was repeated incubating AgNP in ultrapure water and in RPMI 1640 medium without FBS. For comparison, the GOAg nanocomposite was incubated under identical conditions. The stability was also visually observed through digital photographs taken of the GO, AgNP, and GOAg in biological media over time.

Dynamic light scattering was used to investigate the agglomeration of the nanomaterials over time. Stock dispersions of pristine GO, pristine AgNP, and GOAg nanocomposite were diluted in DI water, RPMI, and RPMI supplemented with FBS. Then, the hydrodynamic sizes and the polydispersity index were measured shortly after the dispersion and after 24 and 48 h of incubation in a cell culture chamber at 37 °C and pH 7.4.

### Toxicity of GO, AgNP, and GOAg nanocomposite to macrophage cells

#### Tumoral macrophages

Toxicity tests were performed with murine macrophages of a tumoral lineage, J774 (Banco de Células do Rio de Janeiro, Rio de Janeiro, Brazil). The cells were cultured in bottles, and prior to the toxicity test, approximately 1 × 10^5^ macrophages were transferred to 24-well microplates to let cells adhere for 24 h. Next, the cell medium was withdrawn and replaced by aliquots of stock dispersions of AgNP and GOAg diluted in RPMI 1640 medium supplemented with 10 % FBS to reach silver concentrations of 1.25, 2.5, 5.0, and 10 μg mL^−1^. The microplates were incubated at 37 °C with 5 % CO_2_ for 24 and 48 h. After the incubation period, the cells were removed from the microplates using a cell scraper and stained with trypan blue (Sigma-Aldrich). The number of viable cells was counted in a Neubauer chamber with an optical microscope. After exposure to nanomaterials, macrophages adhered to the coverslips were stained according to the Giemsa protocol for a cell morphology survey. The same procedure was conducted for a stock dispersion of GO diluted in RPMI 1640 medium supplemented with 10 % FBS to reach concentrations of 6.25, 12.5, 25, 50, and 100 µg mL^−1^.

#### Peritoneal macrophages

The cells were collected from Balb/c mouse (Multidisciplinary Center for Biological Investigation on Laboratory Animal Science, University of Campinas, Brazil), rinsed with a phosphate-buffered saline (PBS) solution and centrifuged for 10 min (4 °C, 1500 rpm). Approximately 1 × 10^5^ macrophages were transferred to 24-well microplates containing sterile coverslips to let the cells adhere for 2 h. Next, the cells were rinsed with PBS to remove non-adhered cells and cultured in RPMI medium supplemented with 10 % FBS. After 24 h, the cell medium was withdrawn and replaced by aliquots of stock dispersions of AgNP and GOAg diluted in RPMI 1640 medium supplemented with 10 % FBS to reach silver concentrations of 1.25, 2.5, 5.0, and 10 µg mL^−1^. The microplates were incubated at 37 °C with 5 % CO_2_ for 24 and 48 h. After exposure, the peritoneal macrophages were fixed with methanol and stained by Giemsa. The reduction in the population of peritoneal macrophages was determined by counting the number of adhered macrophages using an optical microscope. The same procedure was conducted for a stock dispersion of GO diluted in RPMI 1640 medium supplemented with 10 % FBS to reach concentrations of 6.25, 12.5, 25, 50, and 100 µg mL^−1^.

Control experiments were performed without the addition of nanomaterials for both macrophage cells. All toxicity tests were carried out in triplicate.

Prior any animal experimentation, this study proposal was analyzed and approved by the ethics committee of the Institute of Biology, University of Campinas, Brazil. Protocol N° 3974-1.

### Induction of oxidative stress

The production of reactive oxygen species (ROS) by the macrophages after AgNP and GOAg nanocomposite exposure was determined by a fluorometric intracellular ROS assay kit (MAK 145, Sigma-Aldrich) based on a superoxide fluorescent probe (dihydroethidium). Prior to testing the nanomaterials samples, hydrogen peroxide up to 5.0 mmol L^−1^ (H_2_O_2_, Synth) was used to confirm the kit’s effectiveness for detecting ROS (Additional file 5: Figure S4). J774 tumoral macrophage cells were plated in 96-well black microplates with clear flat bottoms to a cellular concentration of 1 × 10^4^ cells/mL. The cells were then allowed to adhere for 24 h. The ROS reagent was reconstituted with dimethyl sulfoxide followed by dilution in the assay buffer. Shortly afterward, the ROS reagent solution was added to each well and incubated for 1 h. Next, aliquots of stock dispersions of AgNP and GOAg were diluted in RPMI 1640 medium supplemented with 10 % FBS to reach silver concentrations of 0.6, 1.25, 2.5, and 5.0 µg mL^−1^. The microplates were incubated for 30 and 180 min, followed by 24 and 48 h. The fluorescence intensity was measured in a plate reader (Synergy HT, Biotek) with an excitation wavelength of 520 nm and emission wavelength of 605 nm. The data were normalized on the basis of the cellular viability and were shown as relative fluorescence compared to the negative control (without nanomaterials).

### Nanomaterial uptake by macrophages

#### Assessment of nanomaterial internalization

Transmission electron microscopic images of the J774 tumoral macrophages were taken for both the cellular ultrastructural analysis and the assessment of nanomaterial internalization. The macrophages (1 × 10^5^ cells/mL) were plated in 6-well microplates on coverslips. The cells were then allowed to adhere for 24 h. Next, the cell medium was withdrawn and replaced by aliquots of stock dispersions of GO, AgNP, and GOAg diluted in RPMI 1640 medium supplemented with 10 % FBS to reach concentrations of 12.5, 2.5, and 2.5 µg mL^−1^, respectively. The nanomaterials were separately added to the microplates and incubated for 24 h. After the incubation period, the cellular medium was replaced by a fixative solution containing 2.5 % glutaraldehyde (0.1 mol L^−1^), sodium cacodylate buffer (0.1 mol L^−1^, pH 7.4), and calcium chloride (3 mmol L^−1^) for 5 min at room temperature, followed by 1 h in an ice bath. Next, the cells were rinsed with cacodylate buffer/calcium chloride and were post-fixed with 1 % osmium tetroxide, cacodylate buffer (0.1 mol L^−1^), calcium chloride (3 mmol L^−1^), and potassium ferrocyanide solution (0.8 %) for 30 min on ice. After fixation, the cells were washed with DI water and stained with 2 % uranyl acetate overnight at 4 °C. In the following day, the cells were washed with DI water and sequentially dehydrated in an ascending series of ethanol (20, 50, 70, 80 %, and twice at 100 %). The cells were embedded in Epon 812 resin for 72 h and placed in an oven for polymerization at 60 °C. The monolayer culture ultra-thin sections were stained with uranyl acetate and lead citrate, and then transferred to uncoated copper grids and examined in a Zeiss LEO 902 transmission electron microscope at an accelerating voltage of 60 kV.

#### Quantification of internalized silver

Cell culture bottles were seeded with 1 × 10^5^ cells/mL of J774 tumoral macrophages. After 48 h, the RPMI 1640 medium supplemented with 10 % FBS was replaced by aliquots of stock dispersions of AgNP and GOAg diluted in RPMI 1640 medium supplemented with 10 % FBS to reach a silver concentration of 1 μg mL^−1^. The cells were incubated at 37 °C and 5 % CO_2_ for 24 and 48 h. After incubation, the supernatant was collected and the cells were washed three times with a PBS solution to remove non-internalized nanomaterials. The macrophages were carefully transferred to vials containing PBS solution and were centrifuged for 10 min at 1500 rpm (Eppendorf 5804R). Subsequently, the cell pellets were digested in HNO_3_, (65 % v v^−1^, Merck) and suspended in ultrapure water (Milli-Q^®^ purification system). The internalization of the pristine silver nanoparticles and the nanocomposite was estimated by inductively coupled plasma mass spectrometry (ICP-MS, model 7700x Agilent Technologies, Hachioji, Japan). A reaction/collision system with helium gas was used with a flow rate of 5 mL min^−1^. The experiment was performed in triplicate. The Ag^+^ standard solution was purchased from J.T. Baker.

### Statistical analysis

All experiments were performed in triplicate and the data were presented as the mean values ± standard deviation. The OriginLab 8.5 software was used to perform the analysis of variance (ANOVA) and means comparison by the Tukey test. IC_50_ calculations were performed using a sigmoidal fit with a dose–response function.

## Additional files

10.1186/s12951-016-0165-1 UV–Vis absorption spectra of pristine silver nanoparticles (black line) and graphene oxide-silver nanocomposite (blue line).
10.1186/s12951-016-0165-1 X-ray diffraction patterns of graphene oxide (A), pristine silver nanoparticles (B) and graphene oxide-silver nanocomposite (C).
10.1186/s12951-016-0165-1 Photographs of graphene oxide dispersed in deionized water (DI), RPMI, and RPMI + 10 % FBS during 48 h of incubation. Black arrows indicate precipitation of the nanomaterial.
10.1186/s12951-016-0165-1 Summary of the hydrodynamic size and polydispersity index of pristine graphene oxide, pristine silver nanoparticles, and graphene oxide-silver nanocomposite in different media diluent (n = 3, runs = 10).
10.1186/s12951-016-0165-1 Effectiveness of MAK 145 Kit for reactive oxygen species detection in macrophage J774.

## Abbreviations

GO: graphene oxide
AgNP: silver nanoparticle
GOAg: graphene oxide-silver nanocomposite
ROS: reactive oxygen species
ICP OES: inductively coupled plasma optical emission spectrometry
ICP-MS: inductively coupled plasma mass spectrometry
DLS: dynamic light scattering
TEM: transmission electron microscopy
TLR4: Toll-Like receptor 4
AFM: atomic force microscopy
DI: deionized water
FBS: fetal bovine serum
IC_50_: inhibition concentration 50 %
MØ: macrophage
PBS: phosphate-buffered saline
PVDF: polyvinylidene fluoride
