# Supplementary material for: Comparative in vitro toxicity of a graphene oxide-silver nanocomposite and the pristine counterparts toward macrophages
Source: J Nanobiotechnology. 2016 Feb 24;14:12. doi: 10.1186/s12951-016-0165-1 (PMC4765018; doi:10.1186/s12951-016-0165-1)
Supplement: Supplementary file 2 — 10.1186/s12951-016-0165-1 X-ray diffraction patterns of graphene oxide (A), pristine silver nanoparticles (B) and graphene oxide-silver nanocomposite (C). [file 12951_2016_165_MOESM2_ESM.docx]

**Figure S2** X-ray diffraction patterns of graphene oxide (A), pristine silver nanoparticles (B) and graphene oxide-silver nanocomposite (C)


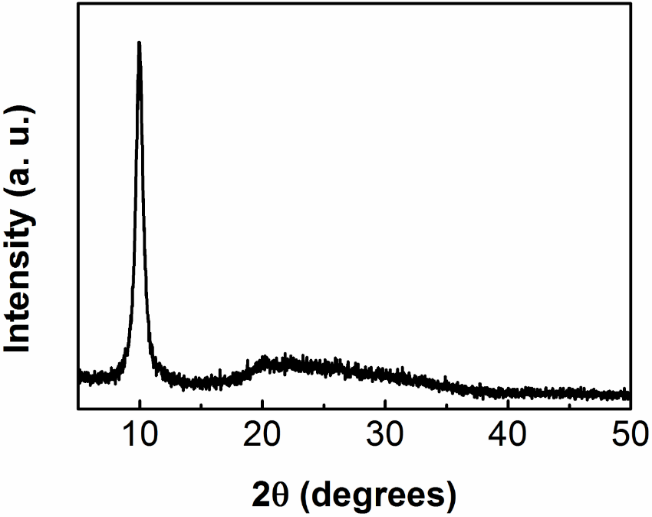

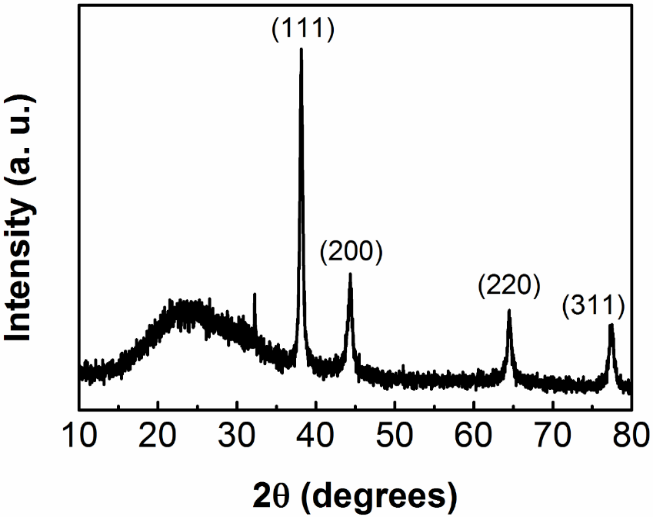

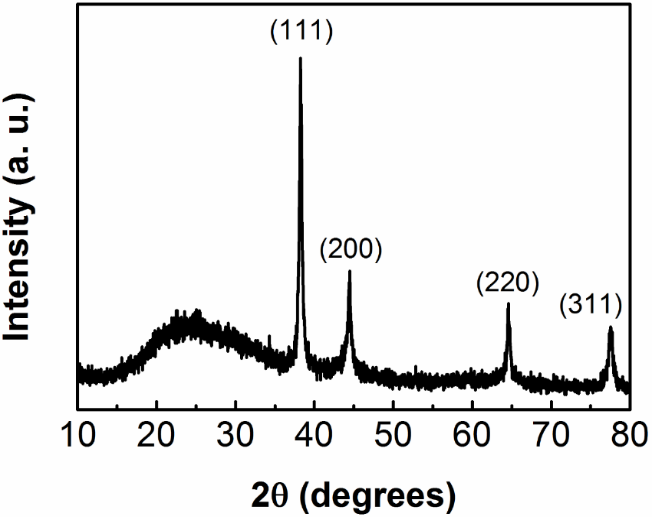


**A**

**B**

**C**

**GO**

**AgNP**

**GOAg**
