# Supplementary material for: Comparative in vitro toxicity of a graphene oxide-silver nanocomposite and the pristine counterparts toward macrophages
Source: J Nanobiotechnology. 2016 Feb 24;14:12. doi: 10.1186/s12951-016-0165-1 (PMC4765018; doi:10.1186/s12951-016-0165-1)

**Figure S3.** Photographs of graphene oxide dispersed in deionized water (DI), RPMI, and RPMI + 10% FBS during 48 h of incubation. Black arrows indicate precipitation of the nanomaterial.

**RPMI**

**0 h**

**1 h**

**3 h**

**6 h**

**12 h**

**24 h**

**GO**

**48 h**


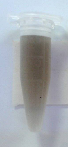

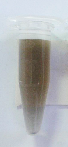

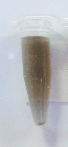

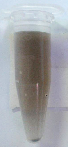

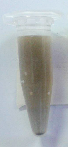

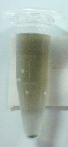


**DI**


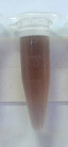

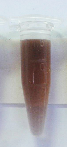

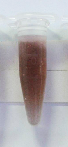

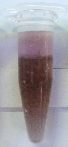

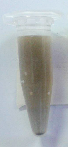

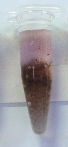

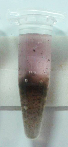

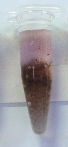


**RPMI +**

**FBS**


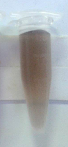

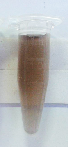

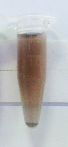

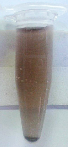

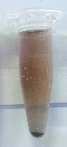

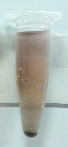

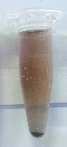

Supplement: Supplementary file 3 — 10.1186/s12951-016-0165-1 Photographs of graphene oxide dispersed in deionized water (DI), RPMI, and RPMI + 10 % FBS during 48 h of incubation. Black arrows indicate precipitation of the nanomaterial. [file 12951_2016_165_MOESM3_ESM.docx]
