# Supplementary material for: Comparative in vitro toxicity of a graphene oxide-silver nanocomposite and the pristine counterparts toward macrophages
Source: J Nanobiotechnology. 2016 Feb 24;14:12. doi: 10.1186/s12951-016-0165-1 (PMC4765018; doi:10.1186/s12951-016-0165-1)
Supplement: Supplementary file 4 — 10.1186/s12951-016-0165-1 Summary of the hydrodynamic size and polydispersity index of pristine graphene oxide, pristine silver nanoparticles, and graphene oxide-silver nanocomposite in different media diluent (n = 3, runs = 10). [file 12951_2016_165_MOESM4_ESM.docx]

**Table S1.** Summary of the hydrodynamic size and polydispersity index of pristine graphene oxide, pristine silver nanoparticles, and graphene oxide-silver nanocomposite in different media diluent (n = 3, runs = 10).

| **GO** | **DLS** | | | | | |
| --- | --- | --- | --- | --- | --- | --- |
|  | **Z-avg (nm)** | | | **PDI** | | |
|  | **0 h** | **24 h** | **48 h** | **0 h** | **24 h** | **48 h** |
| **DI** | 5956 ± 1385 | 5606 ± 744 | 3057 ± 505 | 1.0 | 1.0 | 1.0 |
| **RPMI** | > 10000 | 4917 ± 3014 | 3316 ± 416 | 0.7 | 0.8 | 1.0 |
| **RPMI + FBS** | 4600 ± 2854 | 2105 ± 214 | 1529 ± 543 | 1.0 | 0.9 | 0.8 |
|  | | | | | | |
| **AgNP** | **DLS** | | | | | |
|  | **Z-avg (nm)** | | | **PDI** | | |
|  | **0 h** | **24 h** | **48 h** | **0 h** | **24 h** | **48 h** |
| **DI** | 22.8 ± 1.0 | 27.5 ± 1.1 | 29.3 ± 1.1 | 1.0 | 1.0 | 1.0 |
| **RPMI** | > 1000 | > 1000 | > 1000 | 1.0 | 1.0 | 1.0 |
| **RPMI + FBS** | 33.7 ± 1.0 | 35.5 ± 1.7 | 35.6 ± 0.6 | 0.5 | 0.2 | 0.2 |
|  | | | | | | |
| **GOAg** | **DLS** | | | | | |
|  | **Z-avg (nm)** | | | **PDI** | | |
|  | **0 h** | **24 h** | **48 h** | **0 h** | **24 h** | **48 h** |
| **DI** | 222.1 ± 13.5 | 220.4 ± 2.1 | 152.5 ± 6.6 | 0.4 | 0.3 | 0.4 |
| **RPMI** | 2385 ± 410 | 4647 ± 2071 | 2149 ± 398 | 0.7 | 1.0 | 0.9 |
| **RPMI + FBS** | 197.9 ± 2.9 | 231.4 ± 2.9 | 218.1 ± 9.6 | 0.4 | 0.3 | 0.3 |

DLS: Dynamic light scattering. DI: deionized water. RPMI: Cell media. FBS: Fetal Bovine Serum. Z-avg: average hydrodynamic size. PDI: Polydispersity Index
