# Supplementary material for: Comparative in vitro toxicity of a graphene oxide-silver nanocomposite and the pristine counterparts toward macrophages
Source: J Nanobiotechnology. 2016 Feb 24;14:12. doi: 10.1186/s12951-016-0165-1 (PMC4765018; doi:10.1186/s12951-016-0165-1)
Supplement: Supplementary file 5 — 10.1186/s12951-016-0165-1 Effectiveness of MAK 145 Kit for reactive oxygen species detection in macrophage J774. [file 12951_2016_165_MOESM5_ESM.docx]

**Figure S4.** Effectiveness of MAK 145 Kit for reactive oxygen species detection in macrophage J774.
